# Characteristics of the Intestinal Flora of TPOAb-Positive Women With Subclinical Hypothyroidism in the Second Trimester of Pregnancy: A Single-Center Prospective Cohort Study

**DOI:** 10.3389/fcimb.2022.794170

**Published:** 2022-05-19

**Authors:** Min Wu, Yuxi Yang, Yali Fan, Shan Guo, Tianhe Li, Muqing Gu, Tingting Zhang, Huimin Gao, Ruixia Liu, Chenghong Yin

**Affiliations:** ^1^ Department of Internal Medicine, Beijing Obstetrics and Gynecology Hospital, Capital Medical University, Beijing Maternal and Child Health Care Hospital, Beijing, China; ^2^ Department of Central Laboratory, Beijing Obstetrics and Gynecology Hospital, Capital Medical University, Beijing Maternal and Child Health Care Hospital, Beijing, China

**Keywords:** subclinical hypothyroidism during pregnancy, anti-thyroid peroxidase antibody, intestinal flora, second trimester, levothyroxine

## Abstract

Pregnant women are at high risk of developing subclinical hypothyroidism (SCH), and anti-thyroid peroxidase antibody (TPOAb) positivity can further inhibit thyroxine synthesis. Emerging evidence indicates that intestinal flora can modulate metabolic and immune homeostasis. The characteristics of intestinal flora of TPOAb-positive women with SCH in their second trimester of pregnancy have not been reported. This single-center prospective observational cohort study investigated gut microbial composition and metabolic function using sequencing of the 16S rRNA gene in fecal samples from 75 TPOAb-positive women with SCH and 90 TPOAb-negative women with SCH during their second trimester of pregnancy. Women were treated with no levothyroxine (LT_4_), low-dose LT_4_ (≤50ug/d), or high-dose LT_4_ (>50ug/d). Taxonomic analysis showed *Firmicutes* and *Bacteroidetes* were the dominant phyla, followed by *Actinobacteria* and *Proteobacteria. Faecalibacterium*, *Bacteroides*, *Prevotella 9*, *Bifidobacterium*, *Subdoligranulum*, *Lachnospira*, and *Megamonas* were the predominant genera. The intestinal flora of TPOAb-positive women with SCH who received no LT_4_ was characterized by bacterial amplicon sequence variants (ASVs)/operational taxonomic units (OTUs) enriched in the genus *Subdoligranulum*. The intestinal flora of TPOAb-positive women with SCH who received low-dose or high-dose LT_4_ were characterized by bacterial ASVs/OTUs depleted of the species *Ruminococcus* sp.*_*or *Bacteroides massiliensis*, respectively. A total of 19 metabolic functions of intestinal flora, mainly involving lipid and amino acid metabolism, discriminated TPOAb-positive and TPOAb-negative women with SCH. Our study suggests that there are differences in the composition and metabolic function of intestinal flora of TPOAb-positive and TPOAb-negative women with SCH treated with different doses of LT_4_ in the second trimester of pregnancy. The findings provide insight into intestinal flora as novel targets for the treatment of TPOAb-positive women with SCH during pregnancy.

## Introduction

Subclinical hypothyroidism (SCH) is diagnosed in patients with normal free thyroxine (FT_4_) levels and mildly elevated thyroid-stimulating hormone (TSH) levels (Alexander et al., 2017). The prevalence of SCH in pregnancy ranges from 4 to 25%, and varies according to trimester-specific reference ranges for FT_4_ and TSH (Springer et al., 2017; Dong and Stagnaro-Green, 2019; Fan et al., 2019). The enzyme thyroid peroxidase (TPO) is responsible for the oxidation and organization of iodine, and for the formation of FT_4_ and free triiodothyronine (FT_3_) (McLachlan and Rapoport, 1992). Although most women with SCH are asymptomatic, SCH during pregnancy may be associated with adverse outcomes, including miscarriage, preterm birth, pre-eclampsia and gestational diabetes, especially in women who are anti-thyroid peroxidase antibody (TPOAb) positive (van den Boogaard et al., 2011; Negro and Stagnaro-Green, 2014; Alexander et al., 2017; Arbib et al., 2017; Korevaar et al., 2019). The American Thyroid Association 2017 guidelines recommend assessing TSH levels in women at high risk of thyroid dysfunction, when they are seeking pregnancy or are newly pregnant. Levothyroxine (LT_4_) may be administered to women who are TPOAb-positive with TSH levels higher than the pregnancy-specific reference range, or who are TPOAb-negative with TSH levels > 10.0 mIU/L (Lazarus et al., 2014; Alexander et al., 2017).

In humans, intestinal flora are important for maintaining the integrity of the intestinal mucosal, immune regulation, metabolism, and nutrition (Yang et al., 2016). Intestinal flora can affect the absorption of micronutrients linked to the synthesis and function of thyroid hormones (Virili and Centanni, 2015). Conversely, intestinal flora may interfere with the metabolism and storage of thyroid hormones (Virili and Centanni, 2015). Small intestinal bacterial overgrowth has been reported in patients with overt hypothyroidism; however, the identity of these pathogenic strains of bacteria remains to be elucidated (Lauritano et al., 2007).

To the authors’ knowledge, there are no reports describing the characteristics of intestinal flora of TPOAb-positive women with SCH in their second trimester of pregnancy. In this single-center prospective observational cohort study, we aimed to identify altered gut microbial composition and metabolic function using sequencing of the 16S rRNA gene in fecal samples from 75 TPOAb-positive women with SCH and 90 TPOAb-negative women with SCH during their second trimester of pregnancy. Findings may identify intestinal flora as novel targets for the treatment of TPOAb-positive women with SCH during pregnancy.

## Materials and Methods

### Study Population

This study was a nested prospective observational cohort study that was conducted at Beijing Obstetrics and Gynecology Hospital, Capital Medical University between June, 2020 and March, 2021. This study was approved by the Ethics Committee of the Beijing Obstetrics and Gynecology Hospital (No. 2018-KY-003-01, 2018-KY-003-02). All participants provided written informed consent. The study was registered in the Chinese Clinical Trial Registry (registration number ChiCTR2100047175) on June 10, 2021. All procedures were carried out in accordance with the Declaration of Helsinki.

Inclusion criteria were: (1) females with a singleton pregnancy; (2) recruitment at gestational age 6-13^+6^ weeks; (3) diagnosis of SCH based on thyroid function testing during the first trimester; and (4) provided informed consent.

Exclusion criteria were (1) abortion or loss to follow-up; (2) history of other severe systemic autoimmune disease; (3) history of severe heart, liver, kidney, lung and/or other organ dysfunction; (4) random adjustments to the daily dose of LT4; (5) failure to collect a fecal sample; (6) use of antibiotics or probiotics one month prior to collection of the fecal sample; (7) use of medications that affect thyroid function; (8) history of endemic goiter; or (9) history of mental illness.

### Study Design

Pregnant women were screened to assess thyroid function in the first trimester, according to China’s Guidelines for the Diagnosis and Treatment of Thyroid Diseases Pregnancy and Postpartum (Second Edition), 2019. Serum FT_4_ (enzyme immunoassay), TSH3UL (enzyme immunoassay), and TPOAb levels were measured using an automatic chemiluminescence immunoanalyzer (CENTAUR XP, Siemens, USA). Women’s clinical chemistry or hemoglobin were monitored with an automatic biochemical analyzer (CI16200, Abbott, USA) or blood cell analyzer (XN2000, Sysmex, Japanese), respectively.

TPOAb-positive women with SCH were identified based on TSH3UL>3.56mIU/L, FT4 11.80pmol/L-18.40pmol/L, and TPOAb>60.00U/ml. TPOAb-negative women with SCH were identified based on TSH3UL>3.56mIU/L, FT4 11.80pmol/L-18.40pmol/L, and TPOAb 0.00U/ml-60.00U/ml. TPOAb-positive/negative women with SCH were stratified according to daily dose of LT_4_ during pregnancy: no LT_4_, low-dose LT_4_ (≤50ug/d), or high-dose LT_4_ (>50ug/d).

### Fecal Sample Collection

Fecal samples were collected at 20-23^+6^ weeks of gestation using the PSP^®^ Spin Stool DNA Plus Kit (SARSTEDT, Germany). Pregnant women collected their fecal samples in clean plastic bags after urination. Duplicate samples from the middle of the stool were placed into preservative in individual sterile tubes. Fecal samples were transported to the hospital on the day they were collected and stored at -80°C until analysis.

### High Throughput 16S rRNA Amplicon Sequencing and Analysis

The hypervariable V3-V4 regions of the bacterial 16S rRNA genes were amplified using the following primers: 341F (5′-CCTAYGGGRBGCASCAG-3′) and 806R (5′-GGACTACNNGGGTATCTAAT-3′). Total genomic DNA was extracted using the sodium dodecyl sulfate [SDS] and cetyltrimethyl ammonium bromide [CTAB] methods, according to the manufacturer (TaKaRa MiniBEST Bacterial Genomic DNA Extraction Kit)’s instructions. All PCR reactions were carried out under the following conditions: 15 µL of Phusion^®^High-Fidelity PCR Master Mix (New England Biolabs), 0.2 µM of forward and reverse primers, and approximately 10 ng template DNA. Thermal cycling consisted of initial denaturation at 98°C for 1 min, followed by 30 cycles of denaturation at 98°C for 10 s, annealing at 50°C for 30 s, extension at 72°C for 30 s, and final elongation at 72°C for 5 min. PCR products were detected on 2% agarose gel electrophoresis. PCR products were mixed in equidensity ratios and purified using the Qiagen Gel Extraction Kit (Qiagen, Germany), according to the manufacturer’s instructions. Sequencing libraries were generated using the TruSeq^®^ DNA PCR-Free Sample Preparation Kit (Illumina, USA), according to the manufacturer’s instructions, and index codes were added. Library quality was assessed on the Qubit^®^2.0 Fluorometer (Thermo Scientific) and Agilent Bioanalyzer 2100 system. The library was sequenced on an Illumina NovaSeq platform, and 250 bp paired-end reads were generated.

Microbiome bioinformatics were performed with QIIME2 (2021.04) (Bolyen et al., 2019). Sequences were quality filtered, denoised, merged, and chimeras were removed using the DADA2 plugin (Callahan et al., 2016). Species annotation was performed using QIIME2. 16S annotation was performed using the Silva Database (Release138, http://www.arb-silva.de) (Quast et al., 2013). Alpha diversity indices (Chao1, Shannon, Simpson, Abundance-based Coverage Estimator [ACE]) were calculated with QIIME2 and displayed with R software (Version 3.6.2). Beta diversity was calculated using unweighted unifrac with QIIME2. Principal Coordinate Analysis (PCoA) was performed to visualize similarities and differences in data. A matrix of unweighted unifrac distances was transformed into a new set of orthogonal axes, where the maximum variation factor was demonstrated by the first principal coordinate (PCoA1), and the second maximum variation factor was demonstrated by the second principal coordinate (PCoA2). The two-dimensional PCoA results were displayed using the ade package and ggplot2 package in R (Version 3.6.2). The linear discriminant analysis effect size (LEfSe) (http://huttenhower.sph.harvard.edu/galaxy/; Segata et al., 2011) (LDA score threshold: 2 for functional prediction or 4 for microbiome taxa) was used for quantitative analysis of biomarkers. Functional profiling was performed with Phylogenetic Investigation of Communities by Reconstruction of Unobserved States (PICRUSt2) (https://github.com/picrust/picrust2; Douglas et al., 2020) with a single script (PICRUSt2_pipeline. Py).

### Statistical Analysis of Clinical Data

EpiData was used for data entry, with double data entry and validation. SPSS 26.0 software was used for statistical analysis of clinical data. Normally distributed continuous variables were reported as mean ± standard deviation, and were compared using the independent samples Student’s t test. Non-normally distributed continuous variables were reported as medians and quartiles, and were compared using the Wilcoxon signed-rank test. Categorical variables were reported as frequency [n (%)]. The Wilcoxon signed-rank test was used to compare rank categorical variables. The chi-square test was used when there was no rank association between categorical variables. *P*<0.05 was considered a statistically significant difference.

## Results

### Clinical Characteristics of Subjects

A total of 75 TPOAb-positive women with SCH and 90 TPOAb-negative women with SCH during pregnancy were included in this study. Compared to TPOAb-negative women with SCH, TPOAb-positive women with SCH were more likely to have a history of thyroid disease (41.3% vs. 14.4%, *P*<0.0001) and had significantly higher total cholesterol in the first trimester (4.34mmol/L vs. 4.06mmol/L, *P*=0.022). Other clinical characteristics were not significantly different between groups (**Table 1**).

**Table 1:** Demographic and clinical characteristics of the study participants.

Characteristic	TPOAb-positive women with SCH (n = 75)	TPOAb-negative women with SCH (n = 90)	*P* value
**General information**			
Han ethnicity, *n* (%)	69 (92.0)	81 (90.0)	0.656
Education background (postgraduate and above), *n* (%)	16 (21.3)	21 (23.3)	0.846
Education background (undergraduate), *n* (%)	42 (56.0)	45 (50.0)	
Education background (college and below), *n* (%)	17 (22.7)	24 (26.7)	
Family income (over 4×10^5^yuan/year), *n* (%)	22 (29.3)	25 (27.8)	0.925
Family income (10^5^to 4×10^5^yuan/year), *n* (%)	44 (58.7)	55 (61.1)	
Family income (less than 10^5^ yuan/year), *n* (%)	9 (12.0)	10 (11.1)	
first pregnancy, *n* (%)	41 (54.7)	52 (57.8)	0.688
Thyroid disease history, *n* (%)	31 (41.3)	13 (14.4)	**0.000**
Natural pregnancy, *n* (%)	73 (97.3)	86 (95.6)	0.544
Smoking, *n* (%)	4 (5.3)	6 (6.7)	0.721
drinking, *n* (%)	5 (6.7)	4 (4.4)	0.531
**Indicator in the first trimester**			
Sickness, *n* (%)	25 (33.3)	41 (45.6)	0.111
Animals exposure, *n* (%)	11 (14.7)	16 (17.8)	0.591
Age (year), median (IQR)	33 (30-37)	33 (31-34)	0.623
BMI (kg/m^2^), median (IQR)	21.6 (20.1-24.6)	21.7 (19.9-25.3)	0.961
SBP (mmHg), median (IQR)	112 (104-120)	110 (101-117)	0.153
DBP (mmHg), mean ± SD	67 ± 11	65 ± 9	0.193
ALT (U/L), median (IQR)	12.20 (9.70-20.60)	12.30 (9.78-17.43)	0.766
AST (U/L), median (IQR)	14.40 (13.00-17.30)	15.05 (12.68-16.93)	0.889
ALB (g/L), mean ± SD	43.96 ± 2.50	43.45 ± 1.91	0.188
GLU (mmol/L), median (IQR)	4.63 (4.49-4.93)	4.65 (4.45-4.84)	0.496
BUN (mmol/L), mean ± SD	3.11 ± 0.69	2.96 ± 0.56	0.116
UA (µmol/L), median (IQR)	214.30 (186.00-255.30)	213.70 (183.05-252.48)	0.973
CRE (µmol/L), mean ± SD	49.37 ± 6.76	48.70 ± 5.76	0.491
TC (mmol/L), median (IQR)	4.34 (3.84-4.85)	4.06 (3.74-4.54)	**0.022**
TG (mmol/L), median (IQR)	1.02 (0.70-1.40)	1.00 (0.78-1.44)	0.855
HDL-C (mmol/L), median (IQR)	1.54 (1.33-1.76)	1.42 (1.25-1.59)	0.093
LDL-C (mmol/L), median (IQR)	2.28 (1.93-2.79)	2.10 (1.89-2.46)	0.064
HGB (g/L), mean ± SD	130 ± 10	129 ± 10	0.572

IQR, interquartile range; TPOAb, thyroid peroxidase antibody; BMI, body mass index; SBP, systolicblood pressure; DBP, diastolic blood pressure; ALT, Alanine aminotransferase; AST, Aspartic acid aminotransferase; ALB, albumin; GLU, blood glucose; BUN, blood urea nitrogen; UA, uric acid; CRE, creatinine; TC, total cholesterol; TG, triglycerides; HDL-C, high-density lipoprotein-cholesterol; LDL-C, low-density lipoprotein-cholesterol; HGB, hemoglobin. P<0.05 was considered a statistically significant difference.

After stratifying according to daily dose of LT_4_ during pregnancy, 12 TPOAb-positive women with SCH and no LT_4_ (A1), 24 TPOAb-positive women with SCH and low-dose LT_4_ (A2), 39 TPOAb-positive women with SCH and high-dose LT_4_ (A3), 30 TPOAb-negative women with SCH and no LT_4_ (B1), 43 TPOAb-negative women with SCH and low-dose LT_4_ (B2), and 17 TPOAb-negative women with SCH and high-dose LT_4_ (B3). Microbiome bioinformatic information was compared in TPOAb-positive women with SCH who received no LT_4_ (A1) and TPOAb-negative women with SCH who received no LT_4_ (B1), TPOAb-positive women with SCH who received low-dose LT_4_ (A2) and TPOAb-negative women with SCH who received low-dose LT_4_ (B2), and TPOAb-positive women with SCH who received high-dose LT_4_ (A3) and TPOAb-negative women with SCH who received high-dose LT_4_ (B3).

### Composition of Intestinal Flora

**Figure 1:**
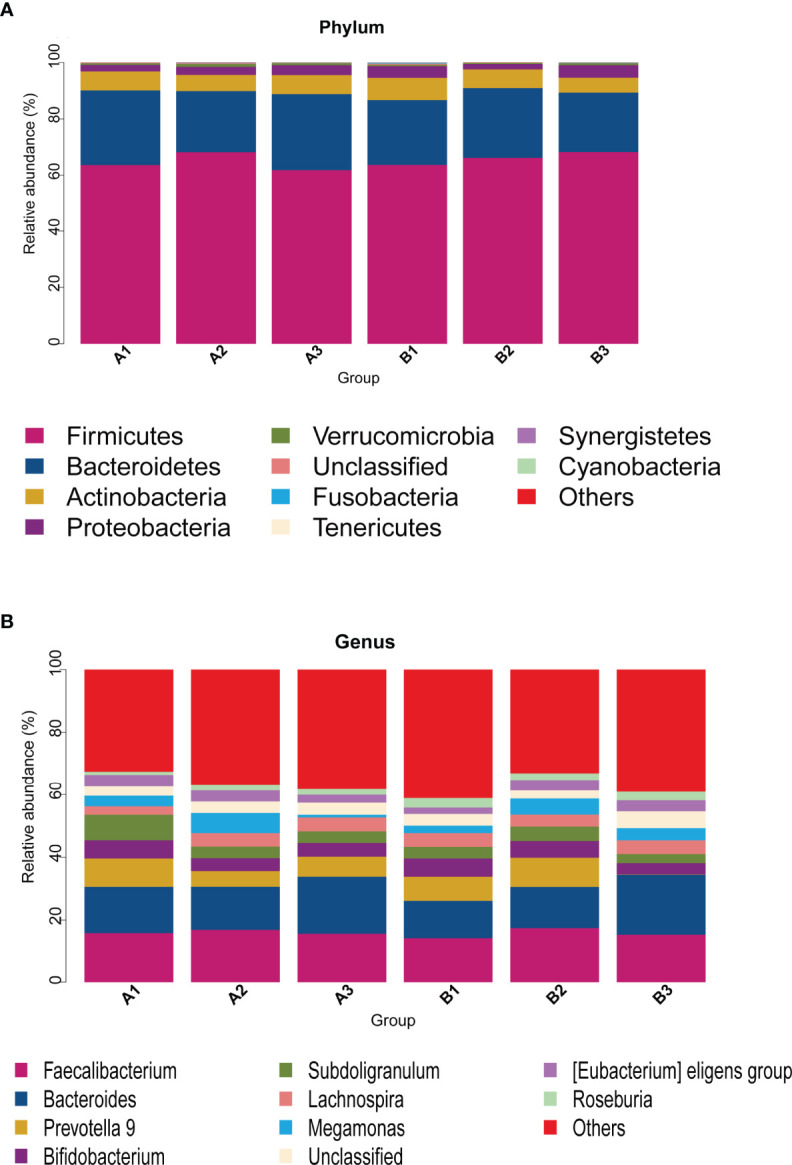
**(A, B)** Taxonomic analysis at the phylum or genus level. A1, TPOAb-positive women with SCH and no LT_4_; A2, TPOAb-positive women with SCH and low-dose LT_4_; A3, TPOAb-positive women with SCH and high-dose LT_4_; B1, TPOAb-negative women with SCH and no LT_4_; B2, TPOAb-negative women with SCH and low-dose LT_4_; B3, TPOAb-negative women with SCH and high-dose LT_4_.

**Figure 2:**
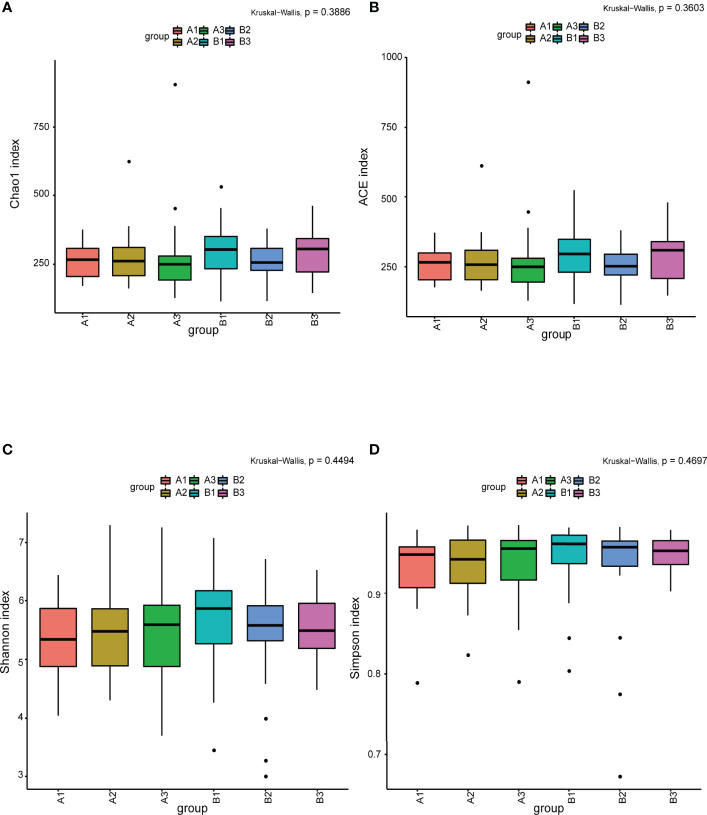
**(A–D)** The α-diversity indices (Chao1, ACE, Shannon, Simpson) analysis. A1, TPOAb-positive women with SCH and no LT_4_; A2, TPOAb-positive women with SCH and low-dose LT_4_; A3, TPOAb-positive women with SCH and high-dose LT_4_; B1, TPOAb-negative women with SCH and no LT_4_; B2, TPOAb-negative women with SCH and low-dose LT_4_; B3, TPOAb-negative women with SCH and high-dose LT_4_.

Among 165 fecal samples, the effective sequence number was 12,357,737. A total of 8,905,780 high-quality reads were identified after sequence denoising or clustering. The number of total/unique/common amplicon sequence variants/operating taxonomic units (ASVs/OTUs) are shown in **Supplementary Figure 1A**. The number of ASVs/OTUs classified to the family, genus and species levels are shown in **Supplementary Figure 1B**. Taxonomic analysis showed *Firmicutes* and *Bacteroidetes* were the dominant phyla, followed by *Actinobacteria* and *Proteobacteria* (**Figure 1A**). *Faecalibacterium*, *Bacteroides*, *Prevotella 9*, *Bifidobacterium*, *Subdoligranulum*, *Lachnospira* and *Megamonas* were the predominant genera (**Figure 1B**). There were no differences in the α-diversity indices between TPOAb-positive and TPOAb-negative women with SCH who received no LT_4_, low-dose LT_4,_ or high-dose LT_4_ (**Figure 2** and **Supplementary Table 1**).

**Figure 3:**
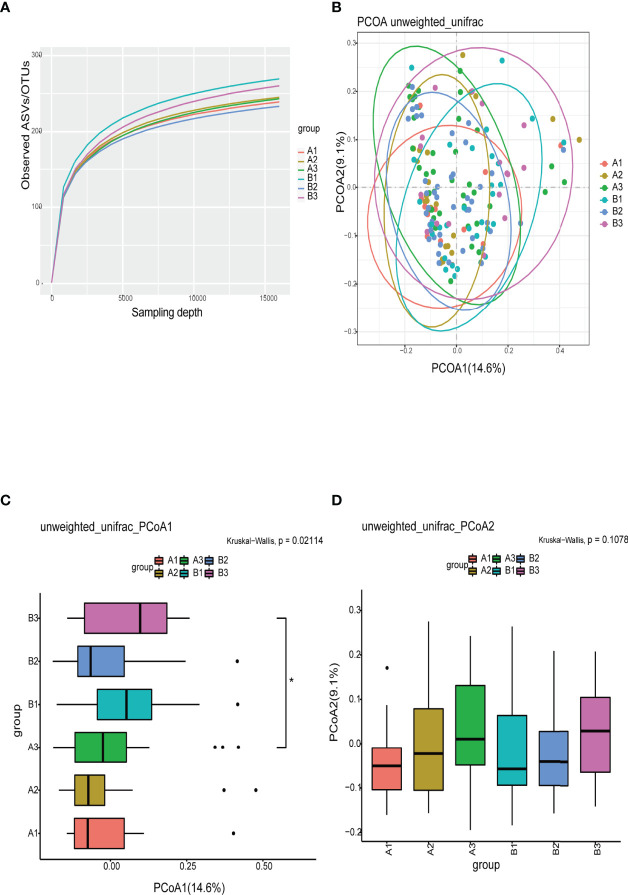
**(A)** Refraction curves based on random extraction of sequencing data from fecal samples from the six subgroups. **(B–D)** β-diversity analysis conducted with the unweighted unifrac algorithm. Ellipses represent 95% confidence intervals for each subgroup in **Figure 3B**. *P<0.05. A1, TPOAb-positive women with SCH and no LT_4_; A2, TPOAb-positive women with SCH and low-dose LT_4_; A3, TPOAb-positive women with SCH and high-dose LT_4_; B1, TPOAb-negative women with SCH and no LT_4_; B2, TPOAb-negative women with SCH and low-dose LT_4_; B3, TPOAb-negative women with SCH and high-dose LT_4_.

The refraction curve of intestinal flora indicated the number of ASVs/OTUs analyzed was sufficient, and the distribution and abundance of species in each subgroup was high and adequate for data analysis (**Figure 3A**). On β-diversity, PCoA1 and PCoA2 explained 14.6% and 9.1% of the observed variation in the taxonomic profiles of intestinal flora across subgroups. The β-diversity of the intestinal flora in TPOAb-positive and TPOAb-negative women with SCH who received high-dose LT_4_ was significantly different (PCoA1, *P*=0.047) (**Figures 3B–D** and **Supplementary Tables 2, 3**). There were no differences in the β-diversity of intestinal flora between TPOAb-positive and TPOAb-negative women with SCH who received no LT_4_ or low-dose LT_4_ (**Figures 3B–D**).

### Intestinal Flora as Markers of SCH During Pregnancy

**Figure 4:**
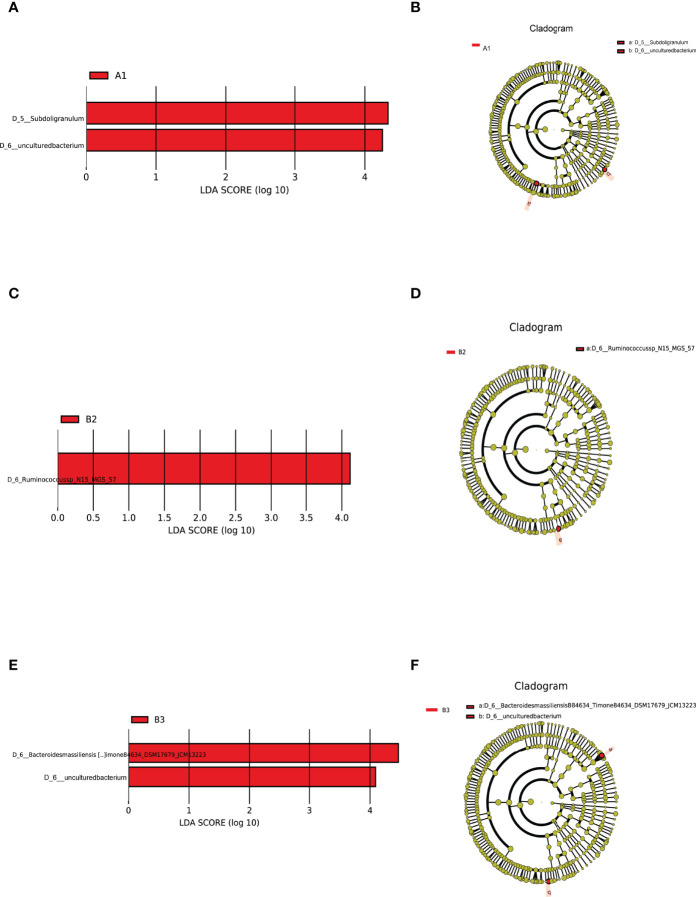
Intestinal flora as markers in TPOAb-positive women with SCH. LEfSe analysis of differential species abundance. **(A, B)** TPOAb-positive vs. TPOAb-negative women with SCH and no LT_4_; **(C, D)** TPOAb-positive vs. TPOAb-negative women with SCH and low-dose LT_4_; **(E, F)** TPOAb-positive vs. TPOAb-negative women with SCH and high-dose LT_4_. LDA value distribution histogram: red bars indicate higher abundance of intestinal flora. Cladograms: circles radiating from the inside to the outside represent taxonomic levels from phylum to species. A1, TPOAb-positive women with SCH and no LT_4_; A2, TPOAb-positive women with SCH and low-dose LT_4_; A3, TPOAb-positive women with SCH and high-dose LT_4_; B1, TPOAb-negative women with SCH and no LT_4_; B2, TPOAb-negative women with SCH and low-dose LT_4_; B3, TPOAb-negative women with SCH and high-dose LT_4_.

LEfSe analysis of differential species abundance was applied to identify intestinal flora that served as markers to distinguish TPOAb-positive and TPOAb-negative women with SCH during pregnancy. The intestinal flora of TPOAb-positive women with SCH who received no LT_4_ was characterized by bacterial ASVs/OTUs enriched in the genus *Subdoligranulum* (**Figures 4A, B** and **Supplementary Table 4**). The intestinal flora of TPOAb-positive women with SCH who received low-dose LT_4_ was characterized by bacterial ASVs/OTUs depleted of the species *Ruminococcussp_N15_MGS_57* (**Figures 4C, D** and **Supplementary Table 4**). The intestinal flora of TPOAb-positive women with SCH who received high-dose LT_4_ was characterized by bacterial ASVs/OTUs depleted of the species *Bacteroides massiliensis B84634_Timone84634_DSM17679_JCM13223* (**Figures 4E, F** and **Supplementary Table 4**). Three marker bacteria (genus *Subdoligranulum*, species *Ruminococcussp_N15_MGS_57* and *Bacteroides massiliensis B84634_Timone84634_DSM17679_JCM13223*) were uniformly distributed among the subgroups (**Supplementary Figures 2A, B** and **Supplementary Table 6**).

### Functional Prediction

**Figure 5:**
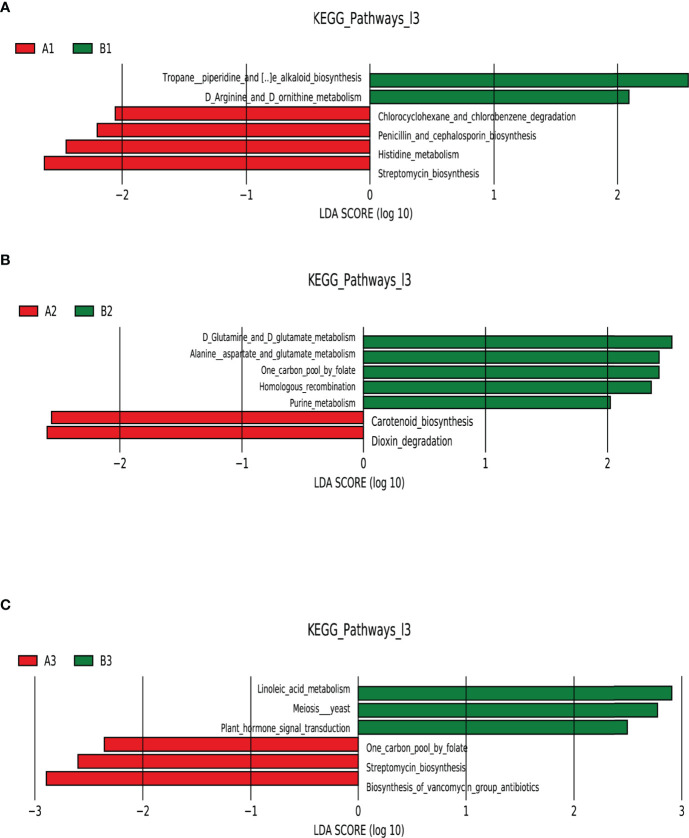
Functional profiling performed with PICRUSt2. LEfSe analysis of differential functional abundance based on the KEGG pathway map. **(A)** TPOAb-positive vs. TPOAb-negative women with SCH and no LT_4_; **(B)** TPOAb-positive vs. TPOAb-negative women with SCH and low-dose LT_4_; **(C)** TPOAb-positive vs. TPOAb-negative women with SCH and high-dose LT_4_. LDA value distribution histogram: the red and green bars represented metabolic functions with higher abundance. A1, TPOAb-positive women with SCH and no LT_4_; A2, TPOAb-positive women with SCH and low-dose LT_4_; A3, TPOAb-positive women with SCH and high-dose LT_4_; B1, TPOAb-negative women with SCH and no LT_4_; B2, TPOAb-negative women with SCH and low-dose LT_4_; B3, TPOAb-negative women with SCH and high-dose LT_4_.

LEfSe analysis of functional abundance based on the KEGG pathway map was used to predict the metabolic functions of the intestinal flora that served as markers to distinguish TPOAb-positive and TPOAb-negative women with SCH during pregnancy. A total of 19 metabolic functions of intestinal flora discriminated TPOAb-positive and TPOAb-negative women with SCH. The intestinal flora of TPOAb-positive women with SCH who received no LT_4_ was characterized by four enriched metabolic functions (including Histidine metabolism) and two depleted metabolic functions (including Arginine and Ornithine metabolism) (**Figure 5A** and **Supplementary Table 5**). The intestinal flora of TPOAb-positive women with SCH who received low-dose LT_4_ was characterized by two enriched metabolic functions and five depleted metabolic functions (including Alanine, Aspartate and Glutamate metabolism) (**Figure 5B** and **Supplementary Table 5**). The intestinal flora of TPOAb-positive women with SCH who received high-dose LT_4_ was characterized by three enriched metabolic functions and three depleted metabolic functions (including Linoleic acid metabolism) (**Figure 5C** and **Supplementary Table 5**).

## Discussion

In this single-center prospective cohort study, we described the composition and characterized the metabolic function of intestinal flora from TPOAb-positive women with SCH in the second trimester of pregnancy. To our knowledge, this is the first study to show that women diagnosed with TPOAb-positive SCH in the first trimester of pregnancy have distinct characteristics of intestinal flora in the second trimester.

In previous reports, non-pregnant female subjects with SCH were more likely to have a family history of thyroid disease than controls (Rafiq-Uddin et al., 2020). Similarly, in this study in pregnant females, TPOAb-positive women with SCH were more likely to have a history of thyroid disease or higher total cholesterol in the first trimester than TPOAb-negative women with SCH. However, total cholesterol of TPOAb-positive and TPOAb-negative women with SCH in the first trimester were within the normal range. Evidence suggests that dietary habits have an effect on gut microbiota composition (Qian et al., 2018). Thus, our study population was selected from Beijing, to minimize the influence of varying dietary habits across regions on intestinal flora. The baseline characteristics that may reflect the dietary habits of the women participating in this study, including ethnic, cultural and economic data, were not significantly different between groups.

The flower chart visually shows the number of common and unique ASVs/OTUs among the six subgroups of women included in this study. Taxonomic analysis revealed similar phyla and genera across the subgroups. α-diversity reflects microbial diversity in a sample; our boxplots showed no differences in the α-diversity indices across subgroups. β-diversity reflects the difference in taxonomic abundance profiles in different samples; We used unweighted unifrac algorithm and PCoA analysis to analyze β-diversity. Boxplots showed significant differences in species diversity at the PCoA1 axis in samples from TPOAb-positive and TPOAb-negative women with SCH who received high-dose LT_4_. Interestingly, we found that median PCoA1 values of TPOAb-positive women with SCH who received no LT_4_, low-dose LT_4_, or high-dose LT_4_ were smaller than those of TPOAb-negative women with SCH who received no LT_4_, low-dose LT_4_, or high-dose LT_4_. In previous studies, immune-mediated diseases, such as type I diabetes (Gianchecchi and Fierabracci, 2017; Mullaney et al., 2018) and systemic lupus erythematosus (SLE) (Corrêa et al., 2017) were associated with a low intestinal microbial diversity (Rooks and Garrett, 2016; Vatanen et al., 2016; Kriss et al., 2018). Consistent with this, we demonstrated low β-diversity (PCoA1) for TPOAb-positive women with SCH.

Harmful or beneficial bacteria may reflect the severity of disease. Based on the results of this study, we can infer that the abundance of the genus *Subdoligranulum* was associated with promoting Histidine metabolism, and inhibiting Arginine and Ornithine metabolism. Thus, the genus *Subdoligranulum* could be harmful bacteria associated with disease progression in TPOAb-positive women with SCH in the second trimester of pregnancy. Conversely, some previous studies revealed that the genus *Subdoligranulum* had beneficial effects on obesity (Dao et al., 2016; Louis et al., 2016); Based on the results of this study, we can also infer that the abundance of the species *Ruminococcussp_N15_MGS_57* (associated with promoting Alanine, Aspartate and Glutamate metabolism) and *Bacteroides massiliensis B84634_Timone84634_DSM17679_JCM13223* (associated with promoting Linoleic acid metabolism) may be beneficial in SCH. Previous studies showed that the genus *Ruminococcus* was a prevalent butyrate-producing gut microbe (Beaud et al., 2005; Takahashi et al., 2016) that has a crucial role in the prevention of metabolic diseases (Arora and Bäckhed, 2016). The species *Bacteroides massiliensis* belongs to the genus *Bacteroides*, which is predominant in the human gut (Wexler and Goodman, 2017). In a previous report, the species *Bacteroides massiliensis* showed an inverse correlation with fecal SARS-CoV-2 load among patients hospitalized with COVID-19 (Zuo et al., 2020). To the authors’ knowledge, our findings are the first to show that the species *Ruminococcussp_N15_MGS_57* and *Bacteroides massiliensis B84634_Timone84634_DSM17679_JCM13223* may have beneficial effects on SCH in the second trimester of pregnancy. Future research should explore the causal relationship between these marker bacteria in the second trimester and TPOAb-positivity in SCH women.

Intestinal flora plays an important role in maintaining the metabolic and immunological balance of the host (Liang et al., 2018). Previous studies have confirmed that there may be a reciprocal interaction between gut microbiota and thyroid disorders (Zhou et al., 2014; Shen et al., 2019; Knezevic et al., 2020; Shin et al., 2020; Su et al., 2020). The mechanisms of intestinal flora in pregnant TPOAb-positive women with SCH remain to be elucidated. Consistent with previous studies that support a role for the molecular mechanisms of intestinal flora in the development of autoimmune diseases (Figura et al., 2019), we identified 19 metabolic functions that discriminate TPOAb-positive and TPOAb-negative women with SCH. The different metabolic functions were mainly involved in lipid and amino acid metabolism.

In conclusion, this single-center prospective cohort study described differences in the composition and metabolic function of intestinal flora between TPOAb-positive and TPOAb-negative women with SCH treated with different doses of LT_4_ in the second trimester of pregnancy. Our data implied that in the second trimester of pregnancy, TPOAb-positive women with SCH possessed a gut microbiome abundant in harmful bacteria (genus *Subdoligranulum*) that regulated amino acid (Histidine, Arginine and Ornithine) metabolism but depleted in two species of beneficial bacteria (species *Ruminococcussp_N15_MGS_57* and *Bacteroides massiliensis B84634_Timone84634_DSM17679_JCM13223*) that promoted amino acid (Alanine, Aspartate and Glutamate) and lipid (Linoleic acid) metabolism, respectively. Although the dietary habits of the pregnant women were not recorded, and their contribution to the gut microbiota of our study population could not be determined, findings provide insights into intestinal flora as novel targets for the treatment of TPOAb-positive women with SCH during pregnancy. Large scale multicenter prospective cohort studies that include dynamic metagenomics and metabolomics analyses are required to verify our findings.

## Data Availability Statement

The datasets presented in this study can be found in online repositories. The names of the repository/repositories and accession number(s) can be found below: https://www.ncbi.nlm.nih.gov/, PRJNA751915.

## Ethics Statement

The studies involving human participants were reviewed and approved by the Ethics Committee of the Beijing Obstetrics and Gynecology Hospital. The patients/participants provided their written informed consent to participate in this study.

## Author Contributions

CY, RL, and MW designed the study. MW, YY, YF, SG, MG, TZ, HG recruited the participants and collected the data and fecal samples. MW and TL performed the microbiological analyses. MW and YY analyzed the data. MW generated the figures and wrote the manuscript. CY and RL critically reviewed and edited the manuscript. All authors agreed to be accountable for all aspects of the work in ensuring that questions related to the accuracy or integrity of any part of the work are appropriately investigated and resolved. All authors read and approved the final manuscript.

## Funding

This work was supported by The National Key Research and Development Program of China (No: 2016YFC1000101) and Supported by Beijing Municipal Science & Technology Commission (No. Z181100001718076). The funders had no role in the study design, data collection and analysis, decision to publish, or preparation of the manuscript.

## Conflict of Interest

The authors declare that the research was conducted in the absence of any commercial or financial relationships that could be construed as a potential conflict of interest.

## Publisher’s Note

All claims expressed in this article are solely those of the authors and do not necessarily represent those of their affiliated organizations, or those of the publisher, the editors and the reviewers. Any product that may be evaluated in this article, or claim that may be made by its manufacturer, is not guaranteed or endorsed by the publisher.

## References

[B1] Alexander E. K. Pearce E. N. Brent G. A. Brown R. S. Chen H. Dosiou C.. (2017). 2017 Guidelines of the American Thyroid Association for the Diagnosis and Management of Thyroid Disease During Pregnancy and the Postpartum. Thyroid: Off. J. Am. Thyroid Assoc. 27 (3), 315–389. doi: 10.1089/thy.2016.0457 28056690

[B2] Arbib N. Hadar E. Sneh-Arbib O. Chen R. Wiznitzer A. Gabbay-Benziv R. (2017). First Trimester Thyroid Stimulating Hormone as an Independent Risk Factor for Adverse Pregnancy Outcome. J. Maternal Fetal Neonatal Med. 30 (18), 2174–2178. doi: 10.1080/14767058.2016.1242123 27677438

[B3] Arora T. Bäckhed F. (2016). The Gut Microbiota and Metabolic Disease: Current Understanding and Future Perspectives. J. Internal Med. 280 (4), 339–349. doi: 10.1111/joim.12508 27071815

[B4] Beaud D. Tailliez P. Anba-Mondoloni J. (2005). Genetic Characterization of the Beta-Glucuronidase Enzyme From a Human Intestinal Bacterium, Ruminococcus Gnavus. Microbiol. (Reading England) 151 (Pt 7), 2323–2330. doi: 10.1099/mic.0.27712-0 16000722

[B5] Bolyen E. Rideout J. R. Dillon M. R. Bokulich N. A. Abnet C. C. Al-Ghalith G. A.. (2019). Reproducible, Interactive, Scalable and Extensible Microbiome Data Science Using QIIME 2. Nat. Biotechnol. 37 (8), 852–857. doi: 10.1038/s41587-019-0209-9 31341288 PMC7015180

[B6] Callahan B. J. McMurdie P. J. Rosen M. J. Han A. W. Johnson A. J. Holmes S. P. (2016). DADA2: High-Resolution Sample Inference From Illumina Amplicon Data. Nat. Methods 13 (7), 581–583. doi: 10.1038/nmeth.3869 27214047 PMC4927377

[B7] Corrêa J. D. Calderaro D. C. Ferreira G. A. Mendonça S. M. Fernandes G. R. Xiao E.. (2017). Subgingival Microbiota Dysbiosis in Systemic Lupus Erythematosus: Association With Periodontal Status. Microbiome 5 (1), 34. doi: 10.1186/s40168-017-0252-z 28320468 PMC5359961

[B8] Dao M. C. Everard A. Aron-Wisnewsky J. Sokolovska N. Prifti E. Verger E. O.. (2016). Akkermansia Muciniphila and Improved Metabolic Health During a Dietary Intervention in Obesity: Relationship With Gut Microbiome Richness and Ecology. Gut 65 (3), 426–436. doi: 10.1136/gutjnl-2014-308778 26100928

[B9] Dong A. C. Stagnaro-Green A. (2019). Differences in Diagnostic Criteria Mask the True Prevalence of Thyroid Disease in Pregnancy: A Systematic Review and Meta-Analysis. Thyroid: Off. J. Am. Thyroid Assoc. 29 (2), 278–289. doi: 10.1089/thy.2018.0475 30444186

[B10] Douglas G. M. Maffei V. J. Zaneveld J. R. Yurgel S. N. Brown J. R. Taylor C. M.. (2020). PICRUSt2 for Prediction of Metagenome Functions. Nat. Biotechnol. 38 (6), 685–688. doi: 10.1038/s41587-020-0548-6 32483366 PMC7365738

[B11] Fan J. Zhang Y. Zhang C. Barjaktarovic M. Yang X. Peeters R. P.. (2019). Persistency of Thyroid Dysfunction From Early to Late Pregnancy. Thyroid: Off. J. Am. Thyroid Assoc. 29 (10), 1475–1484. doi: 10.1089/thy.2019.0115 31347461

[B12] Figura N. Di Cairano G. Moretti E. Iacoponi F. Santucci A. Bernardini G.. (2019). Helicobacter Pylori Infection and Autoimmune Thyroid Diseases: The Role of Virulent Strains. Antibiotics (Basel Switzerland) 9 (1), 12. doi: 10.3390/antibiotics9010012 PMC7167994 31906000

[B13] Gianchecchi E. Fierabracci A. (2017). On the Pathogenesis of Insulin-Dependent Diabetes Mellitus: The Role of Microbiota. Immunol. Res. 65 (1), 242–256. doi: 10.1007/s12026-016-8832-8 27421719

[B14] Knezevic J. Starchl C. Tmava Berisha A. Amrein K. (2020). Thyroid-Gut-Axis: How Does the Microbiota Influence Thyroid Function? Nutrients 12 (6), 1769. doi: 10.3390/nu12061769 PMC7353203 32545596

[B15] Korevaar T. Derakhshan A. Taylor P. N. Meima M. Chen L. Bliddal S.. (2019). Association of Thyroid Function Test Abnormalities and Thyroid Autoimmunity With Preterm Birth: A Systematic Review and Meta-Analysis. JAMA 322 (7), 632–641. doi: 10.1001/jama.2019.10931 31429897 PMC6704759

[B16] Kriss M. Hazleton K. Z. Nusbacher N. M. Martin C. G. Lozupone C. A. (2018). Low Diversity Gut Microbiota Dysbiosis: Drivers, Functional Implications and Recovery. Curr. Opin. Microbiol. 44, 34–40. doi: 10.1016/j.mib.2018.07.003 30036705 PMC6435260

[B17] Lauritano E. C. Bilotta A. L. Gabrielli M. Scarpellini E. Lupascu A. Laginestra A.. (2007). Association Between Hypothyroidism and Small Intestinal Bacterial Overgrowth. J. Clin. Endocrinol. Metab. 92 (11), 4180–4184. doi: 10.1210/jc.2007-0606 17698907

[B18] Lazarus J. Brown R. S. Daumerie C. Hubalewska-Dydejczyk A. Negro R. Vaidya B. (2014). 2014 European Thyroid Association Guidelines for the Management of Subclinical Hypothyroidism in Pregnancy and in Children. Eur. Thyroid J. 3 (2), 76–94. doi: 10.1159/000362597 25114871 PMC4109520

[B19] Liang D. Leung R. K. Guan W. Au W. W. (2018). Involvement of Gut Microbiome in Human Health and Disease: Brief Overview, Knowledge Gaps and Research Opportunities. Gut Pathog. 10, 3. doi: 10.1186/s13099-018-0230-4 29416567 PMC5785832

[B20] Louis S. Tappu R. M. Damms-Machado A. Huson D. H. Bischoff S. C. (2016). Characterization of the Gut Microbial Community of Obese Patients Following a Weight-Loss Intervention Using Whole Metagenome Shotgun Sequencing. PloS One 11 (2), e0149564. doi: 10.1371/journal.pone.0149564 26919743 PMC4769288

[B21] McLachlan S. M. Rapoport B. (1992). The Molecular Biology of Thyroid Peroxidase: Cloning, Expression and Role as Autoantigen in Autoimmune Thyroid Disease. Endoc. Rev. 13 (2), 192–206. doi: 10.1210/edrv-13-2-192 1618162

[B22] Mullaney J. A. Stephens J. E. Costello M. E. Fong C. Geeling B. E. Gavin P. G.. (2018). Correction to: Type 1 Diabetes Susceptibility Alleles are Associated With Distinct Alterations in the Gut Microbiota. Microbiome 6 (1), 51. doi: 10.1186/s40168-018-0438-z 29558994 PMC5859709

[B23] Negro R. Stagnaro-Green A. (2014). Diagnosis and Management of Subclinical Hypothyroidism in Pregnancy. BMJ (Clinical Res. ed.) 349, g4929. doi: 10.1136/bmj.g4929 25288580

[B24] Qian L. Gao R. Hong L. Pan C. Li H. Huang J.. (2018). Association Analysis of Dietary Habits With Gut Microbiota of a Native Chinese Community. Exp. Ther. Med. 16 (2), 856–866. doi: 10.3892/etm.2018.6249 30112040 PMC6090428

[B25] Quast C. Pruesse E. Yilmaz P. Gerken J. Schweer T. Yarza P.. (2013). The SILVA Ribosomal RNA Gene Database Project: Improved Data Processing and Web-Based Tools. Nucleic Acids Res. 41 (Database issue), D590–D596. doi: 10.1093/nar/gks1219 23193283 PMC3531112

[B26] Rafiq-Uddin M. Kamrul-Hasan A. B. Asaduzzaman M. Aminul-Islam A. K. Islam M. Rauniyar B. K.. (2020). Antithyroid Antibody Status in Non-Pregnant Adult Bangladeshi Patients With Subclinical Hypothyroidism. Mymensingh Med. J. MMJ 29 (1), 156–161. 31915352

[B27] Rooks M. G. Garrett W. S. (2016). Gut Microbiota, Metabolites and Host Immunity. Nat. Rev. Immunol. 16 (6), 341–352. doi: 10.1038/nri.2016.42 27231050 PMC5541232

[B28] Segata N. Izard J. Waldron L. Gevers D. Miropolsky L. Garrett W. S.. (2011). Metagenomic Biomarker Discovery and Explanation. Genome Biol. 12 (6), R60. doi: 10.1186/gb-2011-12-6-r60 21702898 PMC3218848

[B29] Shen H. Han J. Li Y. Lu C. Zhou J. Li Y.. (2019). Different Host-Specific Responses in Thyroid Function and Gut Microbiota Modulation Between Diet-Induced Obese and Normal Mice Given the Same Dose of Iodine. Appl. Microbiol. Biotechnol. 103 (8), 3537–3547. doi: 10.1007/s00253-019-09687-1 30850874

[B30] Shin N. R. Bose S. Wang J. H. Nam Y. D. Song E. J. Lim D. W.. (2020). Chemically or Surgically Induced Thyroid Dysfunction Altered Gut Microbiota in Rat Models. FASEB J. Off. Publ. Fed. Am. Societies Exp. Biol. 34 (6), 8686–8701. doi: 10.1096/fj.201903091RR 32356337

[B31] Springer D. Jiskra J. Limanova Z. Zima T. Potlukova E. (2017). Thyroid in Pregnancy: From Physiology to Screening. Crit. Rev. Clin. Lab. Sci. 54 (2), 102–116. doi: 10.1080/10408363.2016.1269309 28102101

[B32] Su X. Zhao Y. Li Y. Ma S. Wang Z. (2020). Gut Dysbiosis is Associated With Primary Hypothyroidism With Interaction on Gut-Thyroid Axis. Clin. Sci. (London England: 1979) 134 (12), 1521–1535. doi: 10.1042/CS20200475 32519746

[B33] Takahashi K. Nishida A. Fujimoto T. Fujii M. Shioya M. Imaeda H.. (2016). Reduced Abundance of Butyrate-Producing Bacteria Species in the Fecal Microbial Community in Crohn's Disease. Digestion 93 (1), 59–65. doi: 10.1159/000441768 26789999

[B34] van den Boogaard E. Vissenberg R. Land J. A. van Wely M. van der Post J. A. Goddijn M.. (2011). Significance of (Sub)Clinical Thyroid Dysfunction and Thyroid Autoimmunity Before Conception and in Early Pregnancy: A Systematic Review. Hum. Reprod. Update 17 (5), 605–619. doi: 10.1093/humupd/dmr024 21622978

[B35] Vatanen T. Kostic A. D. d'Hennezel E. Siljander H. Franzosa E. A. Yassour M.. (2016). Variation in Microbiome LPS Immunogenicity Contributes to Autoimmunity in Humans. Cell 165 (4), 842–853. doi: 10.1016/j.cell.2016.04.007 27133167 PMC4950857

[B36] Virili C. Centanni M. (2015). Does Microbiota Composition Affect Thyroid Homeostasis? Endocrine 49 (3), 583–587. doi: 10.1007/s12020-014-0509-2 25516464

[B37] Wexler A. G. Goodman A. L. (2017). An Insider's Perspective: Bacteroides as a Window Into the Microbiome. Nat. Microbiol. 2, 17026. doi: 10.1038/nmicrobiol.2017.26 28440278 PMC5679392

[B38] Yang I. Corwin E. J. Brennan P. A. Jordan S. Murphy J. R. Dunlop A. (2016). The Infant Microbiome: Implications for Infant Health and Neurocognitive Development. Nurs. Res. 65 (1), 76–88. doi: 10.1097/NNR.0000000000000133 26657483 PMC4681407

[B39] Zhou L. Li X. Ahmed A. Wu D. Liu L. Qiu J.. (2014). Gut Microbe Analysis Between Hyperthyroid and Healthy Individuals. Curr. Microbiol. 69 (5), 675–680. doi: 10.1007/s00284-014-0640-6 24969306

[B40] Zuo T. Zhang F. Lui G. Yeoh Y. K. Li A. Zhan H.. (2020). Alterations in Gut Microbiota of Patients With COVID-19 During Time of Hospitalization. Gastroenterology 159 (3), 944–955.e8. doi: 10.1053/j.gastro.2020.05.048 32442562 PMC7237927

