# Supplementary material for: Characteristics of the Intestinal Flora of TPOAb-Positive Women With Subclinical Hypothyroidism in the Second Trimester of Pregnancy: A Single-Center Prospective Cohort Study
Source: Front Cell Infect Microbiol. 2022 May 19;12:794170. doi: 10.3389/fcimb.2022.794170 (PMC9160305; doi:10.3389/fcimb.2022.794170)
Supplement: Supplementary file 1 [file DataSheet_1.pdf]

SUPPLEMENTARY TABLE 1 | α-diversity indices

α-diversity index	A1_B1_p.value	A2_B2_p.value	A3_B3_p.value
Chao1	0.302932	0.824078	0.280981
ACE	0.253648	0.895960	0.322671
Shannon	0.163888	0.410037	0.562498
Simpson	0.147673	0.373889	0.823735

A1, TPOAb-positive women with SCH and no LT_4; A2, TPOAb-positive women with SCH and low-dose LT_4; A3, TPOAb-positive women with SCH and high-dose LT_4; B1, TPOAb-negative women with SCH and no LT_4; B2, TPOAb-negative women with SCH and low-dose LT_4; B3, TPOAb-negative women with SCH and high-dose LT_4. $P<0.05$ was considered a statistically significant difference.

SUPPLEMENTARY TABLE 2 | Principal coordinate analysis (PCoA1) conducted with the unweighted unifrac algorithm

Group1	Group2	p.value
A1	B1	0.066123
A2	B2	0.353178
A3	B3	**0.046936**

A1, TPOAb-positive women with SCH and no LT_4; A2, TPOAb-positive women with SCH and low-dose LT_4; A3, TPOAb-positive women with SCH and high-dose LT_4; B1, TPOAb-negative women with SCH and no LT_4; B2, TPOAb-negative women with SCH and low-dose LT_4; B3, TPOAb-negative women with SCH and high-dose LT_4. $P<0.05$ was considered a statistically significant difference.

SUPPLEMENTARY TABLE 3 | Principal coordinate analysis (PCoA2) conducted with the unweighted unifrac algorithm

Group1	Group2	p.value
A1	B1	0.676214
A2	B2	0.694837
A3	B3	0.950269

A1, TPOAb-positive women with SCH and no LT_4; A2, TPOAb-positive women with SCH and low-dose LT_4; A3, TPOAb-positive women with SCH and high-dose LT_4; B1, TPOAb-negative women with SCH and no LT_4; B2, TPOAb-negative women with SCH and low-dose LT_4; B3, TPOAb-negative women with SCH and high-dose LT_4. $P<0.05$ was considered a statistically significant difference.

SUPPLEMENTARY TABLE 4 | LEfSe analysis of differential species abundance beween A1 and B1, A2 and B2, A3 and B3

Feature	log(max)	Group	LDA	p.value
D_0__Bacteria.D_1__Firmicutes.D_2__Clostridia.D_3__Clostridiales.D_4__Ruminococcaceae.D_5__Subdoligranulum	4.923682	A1	4.331077	**0.005844**
D_0__Bacteria.D_1__Proteobacteria.D_2__Gammaproteobacteria.D_3__Betaproteobacteriales.D_4__Burkholderiaceae.D_5__Parasutterella.D_6__unculturedbacterium	4.566440	A1	4.252338	**0.007091**
D_0__Bacteria.D_1__Firmicutes.D_2__Negativicutes.D_3__Selenomonadales.D_4__Acidaminococcaceae.D_5__Phascolarctobacterium.D_6__Ruminococcussp_N15_MGS_57	4.654793	B2	4.115964	**0.010950**
D_0__Bacteria.D_1__Firmicutes.D_2__Erysipelotrichia.D_3__Erysipelotrichales.D_4__Erysipelotrichaceae.D_5__ErysipelotrichaceaeUCG_003.D_6__uncultured bacterium	4.750695	B3	4.090399	**0.026209**
D_0__Bacteria.D_1__Bacteroidetes.D_2__Bacteroidia.D_3__Bacteroidales.D_4__Bacteroidaceae.D_5__Bacteroides.D_6__BacteroidesmassiliensisB84634_Timone84634_DSM17679_JCM13223	4.763444	B3	4.467702	**0.047958**

A1, TPOAb-positive women with SCH and no LT_4; A2, TPOAb-positive women with SCH and low-dose LT_4; A3, TPOAb-positive women with SCH and high-dose LT_4; B1, TPOAb-negative women with SCH and no LT_4; B2, TPOAb-negative women with SCH and low-dose LT_4; B3, TPOAb-negative women with SCH and high-dose LT_4. $P<0.05$ was considered a statistically significant difference.

SUPPLEMENTARY TABLE 5 | LEfSe analysis of differential functional abundance beween A1 and B1, A2 and B2, A3 and B3 based on the KEGG pathway map

Feature	log(max)	Group	LDA	p.value
Tropane__piperidine_and_pyridine_alkaloid_biosynthesis	3.508286	B1	2.565456	**0.043572**
Chlorocyclohexane_and_chlorobenzene_degradation	2.788096	A1	2.055611	**0.025922**
Histidine_metabolism	4.163966	A1	2.451475	**0.010422**
Penicillin_and_cephalosporin_biosynthesis	2.769174	A1	2.200494	**0.012217**
D_Arginine_and_D_ornithine_metabolism	2.673901	B1	2.091290	**0.039367**
Streptomycin_biosynthesis	4.275319	A1	2.626528	**0.011288**
Homologous_recombination	4.215960	B2	2.360865	**0.038818**
Carotenoid_biosynthesis	1.211411	A2	2.559018	**0.020485**
D_Glutamine_and_D_glutamate_metabolism	4.365393	B2	2.528276	**0.038818**
One_carbon_pool_by_folate	4.280245	B2	2.423856	**0.009621**
Purine_metabolism	3.962793	B2	2.022170	**0.049821**
Alanine__aspartate_and_glutamate_metabolism	4.296429	B2	2.424944	**0.013955**
Dioxin_degradation	3.132483	A2	2.595310	**0.013505**
Meiosis___yeast	0.203853	B3	2.776641	**0.010314**
One_carbon_pool_by_folate	4.272409	A3	2.356438	**0.033218**
Linoleic_acid_metabolism	3.317597	B3	2.907936	**0.001589**
Plant_hormone_signal_transduction	0.250116	B3	2.498385	**0.033757**
Biosynthesis_of_vancomycin_group_antibiotics	4.361532	A3	2.892761	**0.025329**
Streptomycin_biosynthesis	4.261760	A3	2.600646	**0.013587**

A1, TPOAb-positive women with SCH and no LT_4; A2, TPOAb-positive women with SCH and low-dose LT_4; A3, TPOAb-positive women with SCH and high-dose LT_4; B1, TPOAb-negative women with SCH and no LT_4; B2, TPOAb-negative women with SCH and low-dose LT_4; B3, TPOAb-negative women with SCH and high-dose LT_4. $P<0.05$ was considered a statistically significant difference.

SUPPLEMENTARY TABLE 6 | Differential abundance analysis of taxonomy *Bacteroides massiliensis B84634_Timone84634_DSM17679_JCM13223* between A3 and B3

Sample name	Group	p.value
A402483	A3	**0.037506**
A656520		
A889338		
A717492		
A044884		
A062082		
A686193		
A723787		
A188358		
A793168		
A174676		
A427765		
B025335	B3	
B823300		
B948489		
B640212		
B370055		
B862461		
B007742		
B421431		
B681296		

Sample name only lists the sample that can annotate *Bacteroides massiliensis B84634_Timone84634_DSM17679_JCM13223*; A3, TPOAb-positive women with SCH and high-dose LT_4; B3, TPOAb-negative women with SCH and high-dose LT_4. $P<0.05$ was considered a statistically significant difference.
