# Supplementary material for: Characteristics of the Intestinal Flora of TPOAb-Positive Women With Subclinical Hypothyroidism in the Second Trimester of Pregnancy: A Single-Center Prospective Cohort Study
Source: Front Cell Infect Microbiol. 2022 May 19;12:794170. doi: 10.3389/fcimb.2022.794170 (PMC9160305; doi:10.3389/fcimb.2022.794170)
Supplement: Supplementary file 2 [file DataSheet_2.pdf]

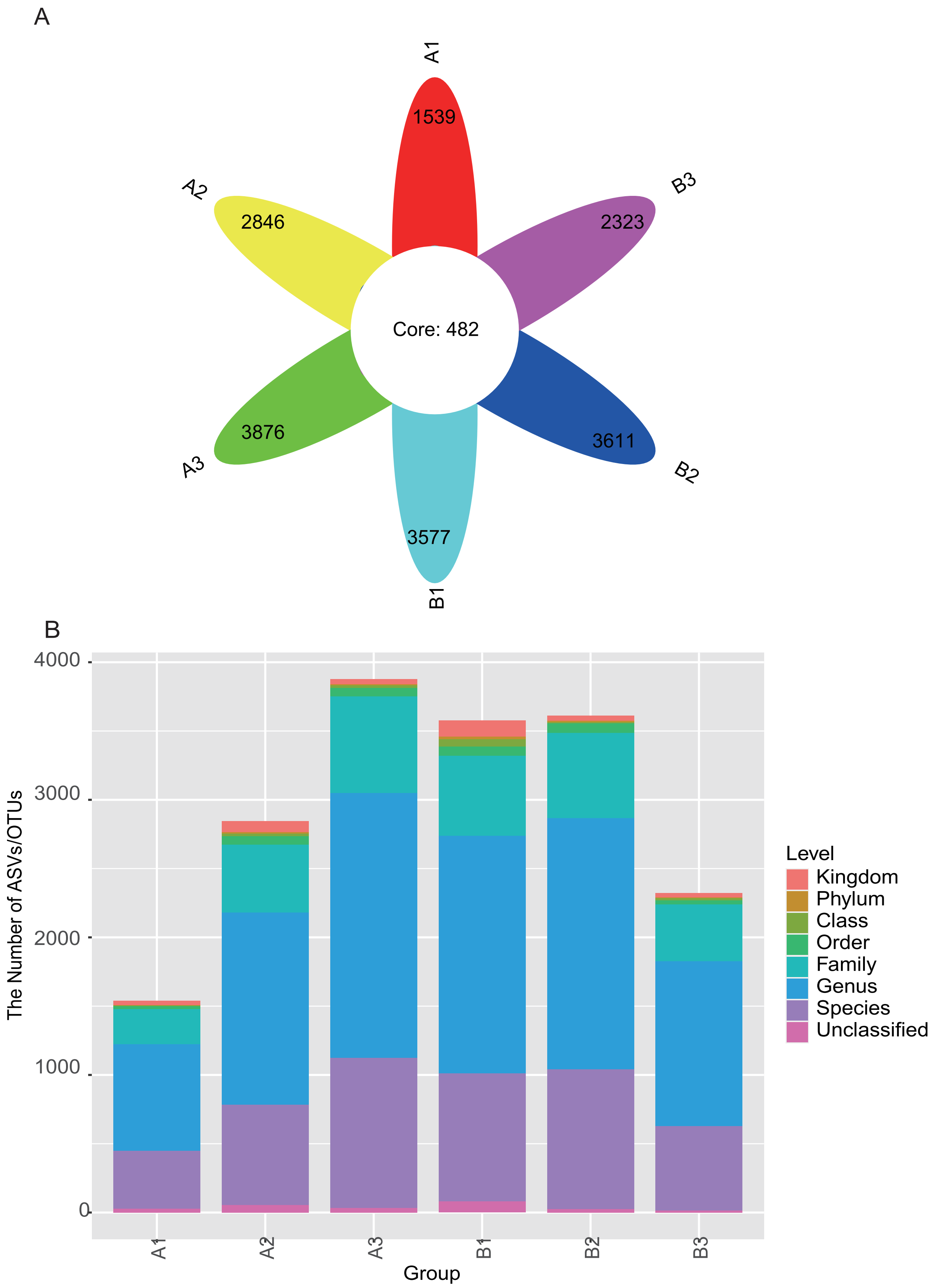

SUPPLEMENTARY FIGURE 1 | (A) Flower diagram showing the number of common and unique ASVs/OTUs among the six subgroups. Different subgroups are represented by different colors. Petals show the ASVs/OTUs unique to each subgroup. The core shows the common ASVs/OTUs across the subgroups. (B) Bar chart showing a large number of ASVs/OTUs can be classified to the family, genus, and species levels.
