# Supplementary material for: Characteristics of the Intestinal Flora of TPOAb-Positive Women With Subclinical Hypothyroidism in the Second Trimester of Pregnancy: A Single-Center Prospective Cohort Study
Source: Front Cell Infect Microbiol. 2022 May 19;12:794170. doi: 10.3389/fcimb.2022.794170 (PMC9160305; doi:10.3389/fcimb.2022.794170)
Supplement: Supplementary file 3 [file DataSheet_3.pdf]

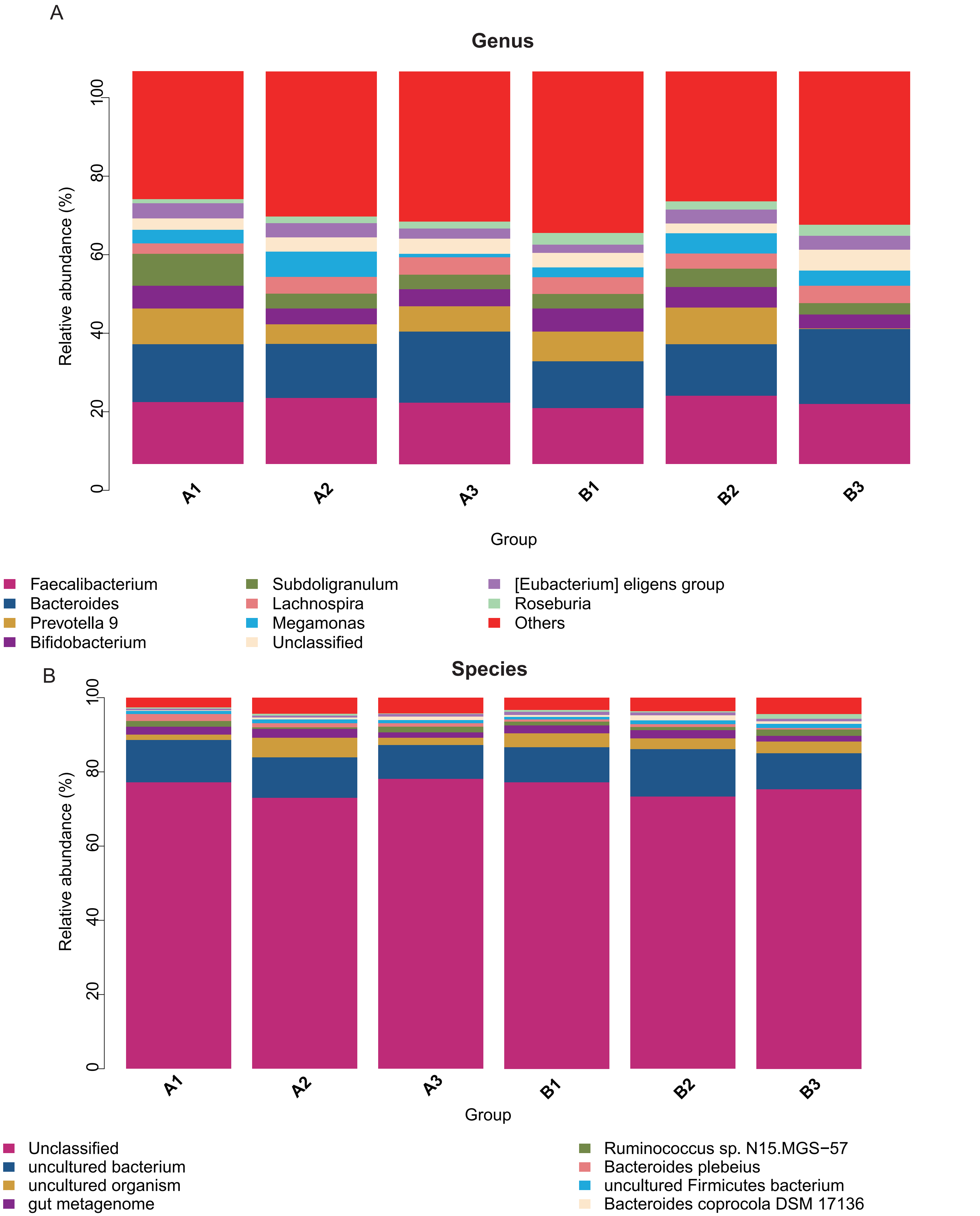

SUPPLEMENTARY FIGURE 2 | (A) Differential abundance of taxonomy *Subdoligranulum* in the six subgroups at the genus level; (B) Differential abundance of taxonomy *Ruminococcussp_N15_MGS_57* in the six subgroups at the species level.
